# Editorial: Contemporary percutaneous interventions for coronary chronic total occlusions

**DOI:** 10.3389/fcvm.2024.1495936

**Published:** 2024-10-02

**Authors:** Iosif Xenogiannis, Antonis N. Pavlidis, Grigorios V. Karamasis

**Affiliations:** ^1^ Department of Cardiology, Attikon University Hospital, Athens, Greece; ^2^ Department of Cardiology, St Thomas’ Hospital, Guy’s and St Thomas’ Hospitals NHS Foundation Trust, London, United Kingdom

**Keywords:** chronic total occlusions, percutaneous coronary interventions, in stent restenosis, rotational atherectomy, collateral vessels, chronic kidney disease, sham procedure, intravascular imaging

**Editorial on the Research Topic**
Contemporary percutaneous interventions for coronary chronic total occlusions

The presence of a chronic total occlusion (CTO), is a common finding among patients who undergo coronary angiography with the estimated prevalence ranging between 16 and 52% for patients with significant (>50 to 70% stenosis) coronary artery disease (CAD) (1, 2). Although the presence of a CTO in coronary angiography has been associated with higher mortality, there are no randomized data showing that successful CTO recanalization can reduce hard clinical endpoints such as mortality or myocardial infarction rate (1, 3). Thus, the main indication of interventional treatment is the control of anginal symptoms. CTO percutaneous coronary intervention (PCI) has high rates of success (80%–90%) when performed by expert on the field operators with a small, but not negligible, rate of complications estimated between 1%–3% (4). Thus, *ad hoc* CTO PCI is discouraged and every patient should be informed in detail about the potential benefits and risks of operation.

The usefulness of CTO recanalization concerning the improvement of left ventricle (LV) systolic function is under debate. A metanalysis of 35 observational studies showed that successful CTO PCI resulted in a, statistically significant, increase in left ventricle ejection function (LVEF) by 3.8% and a reduction in LV end-systolic volume by 4 ml (5). On the contrary, the randomized REVASC study failed to meet its primary endpoint which was the improvement of segmental wall thickening in the territory of the corresponding CTO after PCI. Likewise, LVEF increase was similar in patients who were treated with optimal medical therapy and CTO PCI vs. those who received optimal medical therapy without CTO PCI (6). Yang et al. sought to find predictors of the improvement of LVEF in patients with heart failure and LVEF < 50% undergoing CTO PCI. Improvement in LVEF was defined as an absolute increase of ≥ 10% at 1 year or increase of LVEF up to 40% for patients with baseline LVEF < 40%. Younger age, absence of previous myocardial infarction, smaller left ventricle diastolic diameter and the use of sodium glucose cotransporter 2 inhibitors were all associated with LVEF recovery after CTO PCI. The authors should be complimented for creating a clinically relevant and practical nomogram that will assist physicians to select suitable PCI candidates regarding LVEF improvement after recanalization.

Rotational atherectomy (RA) is among the recommended adjunctive devices that can be used to facilitate PCI (7). It is estimated that rotational atherectomy (RA) is applied in 3.5%–9% of CTO PCIs (8). In general, RA is used for more complex CTO lesions providing high technical success rate with a similar percentage of major adverse cardiovascular events (MACE) compared with cases where RA was not used, but at the expense of higher perforation rates that can reach 10% (8). Tsai et al. compared the clinical outcomes of 44 patients who underwent RA for CTO lesions with 33 propensity matched control patients who were treated with RA for non-CTO lesions. No difference in 30-day MACE was noticed between the two groups (13.5% vs. 13.6%, *p* = 0.987). In addition, there were no significant differences in the incidence of acute slow/no flow, wire transection, vessel perforation, acute heart failure, ventricular arrythmia, acute CIN, reassuring that RA is an effective and safe tool in experienced hands for CTO interventions.

Chronic kidney disease (CKD) has been associated with the presence of advanced atheromatous plaque features and a higher prevalence of coronary calcium (9). Previous studies have shown that patients with CKD who undergo CTO PCI have more comorbidities and more complex lesions. Although technical success is similar compared with cases of CTO PCI in patients without CKD, in-hospital MACE and mortality rate is higher for patients with CKD (10). Zhao et al., dissected 1,076 CTO patients who underwent PCI in four different groups according to eGFR: eGFR ≥ 90 ml/min/1.73 m^2^, 90 > eGFR ≥ 60 ml/min/1.73 m^2^, 60 > eGFR ≥ 30 ml/min/1.73 m^2^ and eGFR < 30 ml/min/1.73 m^2^. In accordance with prior studies, lower eGFR was correlated with a higher number of comorbidities. Interestingly, patients with lower eGFR tended to have fewer “interventional” collaterals. While there was no statistically significant difference in terms of technical success, in-hospital MACE and in-hospital mortality among the four groups, the incidence of pericardiocentesis, major bleeding, and acute renal failure was higher with reduced eGFR and low eGFR was independently associated with an increased risk for in-hospital complications. Finally, whereas successful CTO PCI alleviated symptoms at one month in all four groups this effect waned at one year for patients with eGFR < 30 ml/min/1.73 m^2^. Nevertheless, quality of life, assessed by the EQ-5D questionnaire, improved at a similar degree, regardless renal function, at one month and one year after successful CTO PCI. Thus, patients with advanced renal failure should not be rejected *per se* by interventional cardiologists as they can gain benefit, in terms of quality of life improvement, from CTO recanalization. It should be noted though that patients with e GFR < 30 ml/min/1.73 m^2^ were only a small minority of the total study population (roughly 3%) leading potentially to statistical bias.

It is estimated that in-stent restenosis (ISR) CTOs represent 15% of all CTO PCIs (11). Balloon uncrossable and balloon undilatable lesions (BUs) is the second most common cause of CTO PCI failure only after inability to cross lesion with a guidewire (12). Wang et al. included in their study 218 patients with ISR CTO who underwent PCI of whom 23,9% had BUs. They reported that BUs were associated with a lower rate of technical and procedural success. Importantly, the presence of ostial stents, moderate to severe calcification and moderate to severe tortuosity were all independent predictors of BUs. The previous lesion characteristics in ISR CTOs should alert the interventional cardiologist to be prepared for the upfront use of cutting/scoring balloons or even adjunctive devices such as laser and lithotripsy (13).

Intravascular ultrasound (IVUS) is an extremely useful part of interventionalist's toolbox. In specific for CTO interventions, IVUS has multiple applications: (a) proximal cap ambiguity clarification, (b) antegrade dissection re-entry facilitation, (c) reverse CART facilitation and (d) stent sizing and optimization. Randomized data have shown a reduction in adverse events with the use of IVUS (14, 15). Due to its low penetration depth and inability to image real-time, optical coherence tomography (OCT) has a limited role in CTO PCI. Xenogiannis et al. reviewed in depth the applications of IVUS in CTO interventions providing a practical guide which includes multiple useful “tips and tricks” for the utilization of intravascular imaging in CTO interventions. A variety of clinical cases accompanies the text for a better understating. Furthermore, the most important studies on the field are concisely reported while the role of OCT is also briefly examined.

Collateral vessels are the angiographic trademark of CTOs. The presence of interventional collateral vessels enables the interventional cardiologist to use the retrograde approach if needed, a technique that significantly increased the rates of technical success. PCI of a CTO is recommended when the corresponding myocardial area that is supplied by the totally occluded vessel is viable (4). Previous studies have demonstrated that a well-developed collateral network can predict myocardial viability (16, 17). Liu et al. evaluated the association between hibernating myocardium (HM) and collateral circulation in patients with CTO. Investigators classified patients in two groups according to Rentrop score: patients with poor-developed (Rentrop grades 0–1) and well-developed (Rentrop grades 2–3) collateral circulation. They showed that the higher the HM index (defined as summed rest score in the CTO region −^18^F-FDG uptake score in the CTO region/number of segments with reduced perfusion in the CTO region × 4 × 100%) the higher the likelihood for the presence of well-developed collateral circulation in an approximately linearly positive fashion. Furthermore, the study revealed that patients with well-developed collateral vessels were less likely to have angina, prior myocardial infarction, pathological Q-waves on ECG, left ventricular remodeling and perfusion deficits in the CTO region whereas they had higher LVEF. *Τ*he present study highlights the necessity for thorough viability evaluation in patients with CTO lesions and motion abnormalities of the corresponding myocardial segments. Moreover, we believe that it can pave the way for the development clinically relevant prognostic models for the prediction of LV systolic function recovery after successful CTO PCI.

The Objective Randomized Blinded Investigation with optimal medical Therapy of Angioplasty in stable angina (ORBITA) illustrated the effect of sham intervention in exercise capacity in patients with stable CAD and non-CTO lesions. Since the main goal of CTO PCI is the relief of symptoms, it is easily understood that there is an emerging need for randomized controlled studies (RCTs) evaluating the actual effect of CTO PCI by comparing it with a sham procedure (18). The Sham-Controlled Intervention to improve QOL in CTOs (SHINE-CTO) aspired to address this need, however, it was terminated prematurely due to funding issues during the COVID-19 pandemic. The investigators of the ongoing ISCHEMIA CTO-trial are examining the effect of CTO PCI in two different patient cohorts. In cohort A are included asymptomatic patients with > 10% ischemia of the LV while in cohort B are recruited symptomatic patients with less extensive ischemia (>5%). The primary endpoint in cohort A is the composite of major adverse cardiac and cerebral events and in cohort B the difference in quality of life six months after randomization assessed by Seattle Questionnaire (19).

Khan et al. describe the design as wells as the challenges of ORBITA-CTO trial. The ORBITA-CTO study will be a double-blind, placebo-controlled study of CTO PCI randomizing patients who have: (1) been accepted by a CTO operator for PCI, (2) symptoms attributed to a single-vessel CTO, (3) evidence of ischemia and viability within the CTO territory and (4) J-CTO score ≤ 3 to CTO PCI vs. sham procedure. The primary efficacy outcome is the change in daily ordinal clinical outcome scale, however, the main focus of the trial is to demonstrate the feasibility of a placebo-controlled study in the clinical context of CTO PCI. The data derived from the trial will be used to power a larger study to test the efficacy of CTO PCI vs. placebo in symptomatic CTO patients. As a pilot study, ORBITA-CTO will recruit 50 patients. Symptoms will be assessed using the ORBITA-2 online daily angina symptom application. One of the masin challenges of the study is the correct identification of symptomatic patients. For example, a significant percentage of CAD patients experiences only dyspnea rather than typical anginal symptoms. Moreover, the investigators have to ensure that patients are still symptomatic while they are on optimal anginal therapy. The utilization of the smartphone symptom application is anticipated to increase the fidelity regarding symptom assessment. As it has already been discussed, CTO PCI success rates are high only when performed by operators with expertise in such interventions. Thus, only centers with established CTO operators are eligible to participate in the ORBITA CTO narrowing down the number of potential candidate centers. With the exclusion of lesions with J-CTO score 4 and 5, it is expected that the likelihood of failed cases within the PCI arm will be reduced. CTO interventions are costly, with lower success and higher complications rates compared with non-CTO PCI cases. Hence, the conduction of RCTs that will establish or reject the role of PCI concerning symptomatic benefit of CTO patients are of paramount importance for current clinical cardiology.
